# Supplementary material for: Colorimetric Detection of DNase Type I 3′OH DNA Ends Using an Isothermal Amplification-Assisted Paper-Based Analytical Device
Source: Biosensors (Basel). 2022 Nov 13;12(11):1012. doi: 10.3390/bios12111012 (PMC9688845; doi:10.3390/bios12111012)
Supplement: Supplementary file 1 [file biosensors-12-01012-s001.zip › biosensors-1985511-supplementary.pdf]

Supporting information

Colorimetric Detection of DNase Type I 3′OH DNA Ends Using an Isothermal Amplification-Assisted Paper-Based Analytical Device

Wei Xue, Kaiyun Song, Yangyang Chang, and Meng Liu *

Key Laboratory of Industrial Ecology and Environmental Engineering (Ministry of Education) and Dalian POCT Laboratory, School of Environmental Science and Technology, Dalian University of Technology, Dalian 116024, China

* Correspondence: mliu@dlut.edu.cn

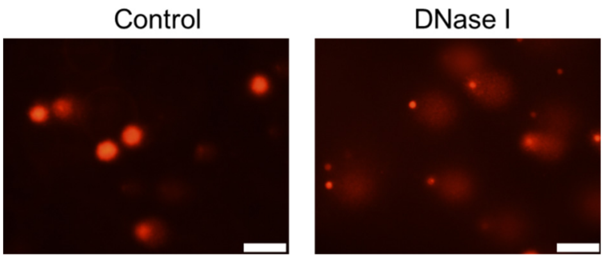

Figure S1. Traditional comet assay for examining DNA damage in ZFL cells. ZFL cells were first treated with or without DNase I at RT for 10 min. Scale bar: 50 μm.

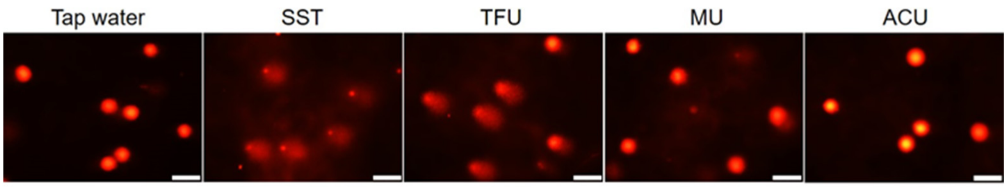

Figure S2. Traditional comet assay. Zebrafish are treated with effluent from different units of AWT. SST: secondary sedimentation tank; TFU: tertiary filtration unit; MU: membrane unit; ACU: activated carbon unit. Scale bar: 50 μm.

Table S1. Comparison of Comet assay, traditional TUNEL and PAD assay for measuring DNA damages.

Comet Assay		TUNEL		PAD	
Slide Preparation	60 min	Fixation	20 min	Cell Lysis	5 min
Lysis	90 min	Permeabilization	10 min	Washing	2 min
Washing	2 min	Washing	2 min	TUNEL	20 min
Electrophoresis	20 min	TUNEL	60 min	Washing	2 min
Neutralization	15 min	Washing	2 min	Cojugation	5 min
DNA Staining	60 min	Cojugation	30 min	Washing	2 min
Imaging	1 min	Washing	2 min	Colorimetry	5 min
		Colorimetry	15 min	Washing	2 min
		Washing	2 min	Imaging	1 min
		Imaging	1 min		
